# Mining the Genome of *Streptomyces leeuwenhoekii*: Two New Type I Baeyer–Villiger Monooxygenases From Atacama Desert

**DOI:** 10.3389/fmicb.2018.01609

**Published:** 2018-07-18

**Authors:** Alejandro Gran-Scheuch, Milos Trajkovic, Loreto Parra, Marco W. Fraaije

**Affiliations:** ^1^Molecular Enzymology Group, University of Groningen, Groningen, Netherlands; ^2^Department of Chemical and Bioprocesses Engineering, School of Engineering, Pontificia Universidad Católica de Chile, Santiago, Chile; ^3^Schools of Engineering, Medicine and Biological Sciences, Institute for Biological and Medical Engineering, Pontificia Universidad Católica de Chile, Santiago, Chile

**Keywords:** Atacama, actinobacteria, Baeyer–Villiger monooxygenase, flavoprotein, biocatalysis

## Abstract

Actinobacteria are an important source of commercial (bio)compounds for the biotechnological and pharmaceutical industry. They have also been successfully exploited in the search of novel biocatalysts. We set out to explore a recently identified actinomycete, *Streptomyces leeuwenhoekii* C34, isolated from a hyper-arid region, the Atacama desert, for Baeyer–Villiger monooxygenases (BVMOs). Such oxidative enzymes are known for their broad applicability as biocatalysts by being able to perform various chemical reactions with high chemo-, regio-, and/or enantioselectivity. By choosing this specific Actinobacterium, which comes from an extreme environment, the respective enzymes are also expected to display attractive features by tolerating harsh conditions. In this work, we identified two genes in the genome of *S. leeuwenhoekii* (*sle_13190 and sle_62070*) that were predicted to encode for Type I BVMOs, the respective flavoproteins share 49% sequence identity. The two genes were cloned, overexpressed in *E. coli* with phosphite dehydrogenase (PTDH) as fusion partner and successfully purified. Both flavin-containing proteins showed NADPH-dependent Baeyer–Villiger oxidation activity for various ketones and sulfoxidation activity with some sulfides. Gratifyingly, both enzymes were found to be rather robust by displaying a relatively high apparent melting temperature (45°C) and tolerating water-miscible cosolvents. Specifically, Sle_62070 was found to be highly active with cyclic ketones and displayed a high regioselectivity by producing only one lactone from 2-phenylcyclohexanone, and high enantioselectivity by producing only normal (-)-1*S*,5*R* and abnormal (-)-1*R*,5*S* lactones (*ee* > 99%) from bicyclo[3.2.0]hept-2-en-6-one. These two newly discovered BVMOs add two new potent biocatalysts to the known collection of BVMOs.

## Introduction

Enzymes are attractive catalysts for several industrial processes by being biodegradable, non-toxic, efficient, and selective. These biocatalysts can offer a high level of safety, low energy consumption, and a global environmentally friendly process (Santiago et al., 2016). Enzyme-based approaches often fulfill all the requirements for ecological and economical viable processes(Dijkman et al., 2013; Reetz, 2013; Woodley, 2017; Pellis et al., 2018). The recognition that enzymes can be used in industrially relevant processes is reflected in a predicted growing market for biocatalysts (Dewan, 2014; Research and Markets, 2017). Particular examples of enzymes that show industrial potential are Baeyer–Villiger monooxygenases (BVMOs). BVMOs are well-studied enzymes (EC. 1.14.13.XX) that can be used for the production of (enantiopure) esters, lactones, and sulfoxides by incorporating an atom of oxygen in an organic substrate releasing a molecule of water using NADPH as cofactor (Bučko et al., 2016). These enzymes typically display a high chemo-, regio-, and enantioselectivity while operating at mild reaction conditions (Leisch et al., 2011). In the last years some new Type I BVMOs have been discovered and characterized from different organisms, such as YMOB (*Yarrowia* monooxygenase) from the yeast *Yarrowia lipolytica*, which shows activity on some ketones and sulfides (Bordewick et al., 2018), the BVMO_AFL706_ and BVMO_AFL334_ from the fungus *Aspergillus flavus*, which showed a broad substrate acceptance including substituted cyclic, aliphatic, and aromatic ketones (Mthethwa et al., 2017), BVMO_Lepto_ from *Leptospira biflexa*, which was used in whole-cell reactions conversions of various ketones (Ceccoli et al., 2014) and PockeMO from *Thermothelomyces thermophila*, which displays a high thermostability and shows activity on bulky substrates (Fürst et al., 2016). However, the BVMOs require special conditions that challenge the application of these biocatalysts on a large scale, like the expensive nicotinamide adenine dinucleotide phosphate (NADPH) as coenzyme. To reduce the costs related to the coenzyme usage, efficient external regeneration systems have been developed. For example, the thermostable phosphite dehydrogenase (PTDH) from *Pseudomonas stutzeri* WM88 can be used to regenerate NAD(P)H (Johannes et al., 2005; Torres Pazmiño et al., 2009). Another major issue concerning the application of BVMOs is the poor stability they often display at industrial conditions, like the presence of cosolvent, high temperature and, in some cases, high salinity (Woodley, 2017; Pellis et al., 2018). Generally, enzymes isolated from mesophilic organisms do not tolerate such conditions. Extremozymes, which are enzymes derived from extremophilic organisms, are typically more suited to withstand harsh environments (Karan et al., 2012). Currently, there is only one BVMO that can tolerate harsh conditions: phenylacetone monooxygenase (PAMO) from *Thermobifida fusca* (Fraaije et al., 2005). This biocatalyst was obtained by a genome mining approach specifically targeting this mesothermophilic actinobacterium. PAMO was found to be rather thermostable while tolerating cosolvents (de Gonzalo et al., 2006; Rodríguez et al., 2008). Recently, other moderately stable BVMOs, isolated from mesothermophilic microbes, were reported (Fürst et al., 2016; Romero et al., 2016). Inspired by these, we considered performing genome mining to another extremophilic actinobacterium to search for novel BVMOs.

The Atacama desert is a hyper-arid area in the north of Chile, characterized by: (a) a large daily temperature variation, where in some areas it ranges from –8 to 50°C (Pulschen et al., 2015); (b) low water availability, the area is considered the driest desert in the world (Azua-Bustos et al., 2012); (c) exposition to high ultraviolet (UV) light, this zone is characterized by its high altitude, prevalent cloudless conditions and relatively low total ozone column, making this desert one of the highest UV radiation sites on Earth (Paulino-Lima et al., 2013; Pulschen et al., 2015) and; (d) high salinity, the desert contains extremely large natural deposits of anions (as Cl, ClO_3_^-^, SO_4_^2-^, ClO_4_^-^, and others). These geographic and environmental characteristics make the microorganisms thriving in the Atacama desert unique, comprising a genetic and molecular treasure that could lead to novel (bio)chemistry (Bull and Asenjo, 2013; Bull et al., 2016; Idris et al., 2017). Several microorganisms have been isolated from the Atacama desert, being an interesting environment to search bacteria with different adaptive qualities to be exploited for biotechnological applications. Among the microorganisms isolated, numerous Actinomycetes have been characterized, including a particular species, *Streptomyces leeuwenhoekii* C34, found to produce novel natural products (Okoro et al., 2009). This actinobacterium is a Gram-positive mycelial bacterium rich in novel pharmaceutical compounds (Busarakam et al., 2014). *S. leeuwenhoekii* was found in a soil sample, grows from 4 to 50°C, optimally at 30°C, from pH 6.0–11, optimally at 7.0, and in the presence of 10% w/v sodium chloride. Because of its highly biotechnological potential, this bacterium was sequenced after its discovery (Gomez-Escribano et al., 2015). Genomic analysis revealed a 72% G+C content, the presence of a linear chromosome (8 Mb) and two extrachromosomal replicons, the circular pSLE1 (86 kb) and the linear pSLE2 (132 kb). The *S. leeuwenhoekii* genome contains 35 gene clusters apparently encoding for the biosynthesis of specialized metabolites with potent antibiotic activity such as chaxamycins and chaxalactins (Rateb et al., 2011; Busarakam et al., 2014). Genome mining in *Streptomyces* isolates has already been reported for the identification and characterization of novel BVMOs, including: (i) MtmOIV, a BVMO isolated from *S. argillaceus* which is a key enzyme in the mithramycin biosynthesis pathway (Gibson et al., 2005), (ii) the BVMOs PenE and PntE forming pentalenolactone precursors in the pathway of antibiotic biosynthesis in *S. exfoliates* and *S. arenae*, respectively (Seo et al., 2011), (iii) two BVMOs from *S. coelicolor* acting on thioanisole and a heptanone (Park et al., 2007), and (iv) PtlE from *S. avermitilis* has also been described to be involved in a pentalenolactone biosynthetic pathway (Jiang et al., 2009). We performed a search in the predicted proteome of *S. leeuwenhoekii* using the sequence motifs described for Type I BVMOs (Fraaije et al., 2002; Riebel et al., 2012). In this work, we report the discovery, expression and characterization of two novel Type I BVMOs fused to PTDH.

## Materials and Methods

### Genome Analysis

The GenomeNet server^1^ was used for searching proteins that harbor specific sequence motifs (Rossmann fold G-x-G-x-x-[GA] and the Type I BVMOs fingerprints [AG]-G-x-W-x-x-x-x-[FY]-[GM]-x-x-x-D and F-x-G-x-x-x-H-x-x-x-W-[PD]) using the predicted proteome of *S. leeuwenhoekii* (code: Actinobacteria, Streptomyces, sle). The UniProt server was used for the identification of the proteins^2^ and the NCBI server for the BLAST searches and identity sequence confirmation ^3^. Multiple sequence alignments were prepared using 45 protein sequences in MUSCLE software (v3.8.31) configured with default settings for highest accuracy and employing the UPGMB clustering method. The phylogenetic tree was reconstructed using the maximum likelihood (ML) method implemented in MEGA 7.0 (500 bootstrap replications). The default substitution model was selected assuming an estimated proportion of invariant sites and 4 gamma-distributed rate categories to account for rate heterogeneity across sites (WAG model). Nearest-Neighbor Interchange (NNI) ML heuristic method was chosen. Initial tree(s) for the heuristic search were obtained by applying the BioNJ method to a matrix of pairwise distances estimated using a JTT model (Whelan and Goldman, 2001; Kumar et al., 2016).

### Reagents, Bacterial Strains, and Plasmids

All chemical reagents were purchased from Sigma-Aldrich, Difco or Merck, unless otherwise stated. Oligonucleotide primers synthesis and DNA sequencing were performed by Macrogen. The genes were amplified by PCR from genomic DNA isolated from *S. leeuwenhoekii* C34. *Escherichia coli* TOP10 (Thermo Fisher Scientific) and *E. coli* NEB 10β (New England Biolabs) were used as host strain for recombinant DNA. The pCRE2 vector was used for expressing the target proteins fused to PTDH equipped with an N-terminal His-tag (Torres Pazmiño et al., 2009). Lysogenic broth (LB), terrific broth (TB), and mannitol soya flour media (SFM) were used for bacterial growth (Hobbs, 1989). PTDH-PAMO (phenylacetone monooxygenase fused to PTDH) and PTDH-TmCHMO (*Thermocrispum municipale* cyclohexanone monooxygenase fused to PTDH) were from GECCO-Biotech.

### Cloning, Expression, and Purification

*Streptomyces leeuwenhoekii* was grown in SFM and its genomic DNA was isolated and purified using Purelink^®^ Genomic DNA kit (Invitrogen) according to the recommendations of the manufacturer. Genes encoding the putative enzymes were amplified by PCR using Phusion High-Fidelity DNA Polymerase with the same program: 95°C-420 s, [95°C-40 s, 59°C-40 s-73°C-120 s] × 35 cycles, 73°C-600 s and 4°C-overnight. For the *sle_13190* gene the forward and reverse primers were: 5^′^-CCT GCG GCT GAC TCG AGA TCT GCA GCT GGT ATG GCC CGC GCC GAA and 5^′^-TTT TGT TCG GGC CCA AGC TTG GTA ATC TAT GTA TCC TGG TCA GCG CAG TTC GAG GCC, respectively. For the *sle_62070* gene the forward and reverse primers were 5^′^-CCT GCG GCT GAC TCG AGA TCT GCA GCT GGT ATG ACA CAA GGT CAG ACG TTG TCC and 5^′^-TTT TGT TCG GGC CCA AGC TTG GTA ATC TAT GTA TCC TGG TCA GCT CAC CGT GGA GCC, respectively. For the *sle_41160* gene the forward and reverse primers were: 5^′^-CCT GCG GCT GAC TCG AGA TCT GCA GCT GGT ATG GCC GAG CAC GAG CAT and 5^′^-TTT TGT TCG GGC CCA AGC TTG GTA ATC TAT GTA TCC TGG TCA CGC GGT CAC CCC. For the pCRE2 amplification the primers were 5^′^-CCA GGA TAC ATA GAT TAC CAA GCT TGG GCC CGA ACA AAA AC and 5^′^-ACC AGC TGC AGA TCT CGA GT. The PCR conditions were optimized to a final concentration of 3% DMSO and 125 nM of each primer. Purified PCR products were cloned into the pCRE2 vector by Gibson assembly (Gibson et al., 2009). Products were used directly for transformation of competent *E. coli* TOP10 cells. Colonies were grown on LB-agar plates supplemented with ampicillin at 37°C. Plasmids were isolated (Wizard^®^Plus SV Minipreps DNA Purification System) and sequenced for cloning confirmation (Macrogen). Verified plasmids were transformed in competent *E. coli* NEB 10β used for protein expression. For purification, a single colony was taken for growing a preculture in LB at 37°C overnight. An aliquot of the preculture (1:100) was used to inoculate fresh TB medium supplemented with 50 μg mL^-1^ ampicillin. Cultures were incubated at 37°C with shaking until an OD_600_ of 0.7 was reached after which expression was induced by adding L-arabinose. To optimize the expression, different inducer concentrations (0.002, 0.02, and 0.2%) and temperatures (17, 24, 30, and 37°C) for 16, 24, and 48 h were tested. Cells were harvested by centrifugation (6,000 × 20^′^ at 4 C using a JLA-9100 rotor, Beckman Coulter) and suspended in lysis buffer (50 mM Tris–HCl pH 7.0, 10% w/v glycerol, 1.5 mg mL^-1^ lysozyme, 10 μM FAD and 1 mM PMSF). Cell extracts (CE) were obtained by sonication (Vibra cell, Sonics, and materials) for 10^′^ (amplitude 70%, 7 s on and 7 s off). The cleared cell extracts (CCEs) were obtained by centrifugation at 10,000 rpm for 1 h at 4°C (Centrifuge 5810R, Eppendorf). CCE, CE, and insoluble fraction were analyzed by SDS-PAGE to verify expression of the respective BVMOs. After establishing proper expression conditions, CCE was prepared, filtered (0.45 μM) and loaded on 3 mL of nickel sepharose HP (GE Health Care) pre-equilibrated with buffer and incubated for 1 h at 4°C in a rotating system. Then, the column was washed with ten column volumes of buffer (50 mM Tris–HCl pH 7.0, 10% glycerol, 0.5 M NaCl) followed by two column volumes of 50 mM Tris–HCl pH 7.0, 10% glycerol, 0.5 M NaCl and 5 mM imidazole. The protein was eluted using buffer with 500 mM imidazole. Fractions containing yellow protein were loaded on a pre-equilibrated Econo-Pac 10DG desalting columns (Bio-Rad). The final sample was flash frozen with liquid nitrogen and stored at –80°C. The purity of each purified enzyme batch was confirmed by SDS-PAGE analysis.

### Fluorescence and Spectrophotometric Analysis

To determine the protein concentration based on FAD content, samples were diluted until an absorbance of around 0.5 at 440 nm. After collecting a full UV-vis spectrum, sodium dodecyl sulfate (SDS) was added to a final concentration of 0.1% w/v. An UV-vis spectrum was recorded again after 10 min. The spectrum obtained with SDS was used to determine the FAD concentration (𝜀 = 11,300 M^-1^ cm^-1^), and the extinction coefficient for the protein by comparison with the spectrum of the native protein (Fraaije et al., 2005).

The apparent melting temperatures (T*_M_*’) were determinate by using the ThermoFAD method (Forneris et al., 2009). For this, 20 μl samples were prepared in a 96-well PCR plate. The samples contained 1 mg mL^-1^ enzyme in different buffered solutions: 50 mM Bis-Tris HCl, 50 mM Tris-HCl, or 10 mM CAPS NaOH buffer adjusted at desired pH, cosolvents, and other additives. The plate was heated from 20 to 99°C, increasing the temperature by 0.5°C every 10 s, using an RT-PCR instrument (CFX96-Touch, Bio-Rad). By measuring fluorescence using a 450–490 nm excitation filter and a 515–530 nm emission filter, the T*_M_*’ or unfolding temperature was determined as the maximum of the derivative of the sigmoidal curve.

Enzyme activity was screened by measuring the oxidation of NADPH at 340 nm (𝜀 = 62,220 M^-1^ cm^-1^) in 96-well F-bottom plates (Greiner Bio-One GmbH) at 25°C using a SynergyMX micro-plate reader (BioTek). The reactions were performed in 200 μL containing 10% glycerol, 50 mM Bis-Tris HCl, 50 mM Tris HCl or 10 mM CAPS at the desired pH, cosolvent concentration, and additive, 0.45 μM of purified BVMO, 150 μM NADPH, 10 μM FAD, and 5.0 mM phenylacetone. As a control, to measure NADPH consumption in the absence of substrate (uncoupling activity), phenylacetone was omitted. For determining kinetic parameters, activity was analyzed using a V-660 spectrophotometer (Jasco) using a 100 μL quartz cuvette at 25°C, and NADPH oxidation at 340 nm was followed. The reaction mixture contained 50 mM Tris-HCl pH 8.0, 10% glycerol, 100 mM NaCl, 0.45 μM of purified BVMO, 150 μM NADPH, 10 μM FAD, and substrate solubilized in 1,4-dioxane (2.5% v/v final concentration). The control reaction contained no substrate and the same concentration of cosolvent. The reaction was started by adding the nicotinamide cofactor and mixing, after which the absorbance was measured for 60 s.

### Biotransformation Studies

The substrate scope analysis was performed using 6 different substrate mixtures (400 μM final concentration of each substrate and 2.5% v/v 1,4-dioxane). For transformations, the reaction mixture was prepared in 500 μL containing Tris–HCl pH 7.0, 10% glycerol, 100 mM NaCl, 30 μM FAD, 10 mM Na_2_PO_3_⋅5H_2_O, 150 μM NADPH, and 2.7 μM of purified enzyme in a 20 mL glass vial. The mixture was subsequently shaken at 150 rpm and 24°C for 24 h. The mixture was extracted three times by mixing one volume of ethyl acetate for 60 s. Subsequently, anhydrous sulfate magnesium was added to the organic solution to remove residual water. Analysis was carried out using a GCMS-QP2010 Ultra (Shimadzu) with electron ionization and quadrupole separation with a HP-1 column. The temperature program, column data and injection volumes are in the Supplementary Table S1.

After identifying the positive substrates and optimizing the conditions, biotransformations with single substrates were carried out in buffer Tris–HCl pH 8.0 using the same concentration for the additives, enzyme, cofactor and phosphite described above. The conversion of racemic bicyclo[3.2.0]hept-2-en-6-one, phenylacetone, 2-phenylcyclohexanone, and 4-phenylcyclohexanone was performed using a final substrate concentration of 5.0 mM. For benzoin, a concentration of 1.0 mM was used. The reaction mixtures were incubated at 24°C and 150 rpm for 2 and 24 h. After incubation, samples were extracted three times with one volume of tert-butyl methyl ether containing 0.1% v/v mesitylene as an external standard and vortexed for 1 min. Then, the GC analysis was performed in the same instrument described above, for chiral analysis of bicyclo[3.2.0]hept-2-en-6-one, 2-phenylcyclohexanone and 4-phenylcyclohexanone the substrates were analyzed using a 7890A GC System (Agilent Technologies) equipped with a CP-Chirasil-Dex CB column. The enantiomers were identified by comparing with reported retention times and biocatalytic preference (Alphand et al., 1996; Vogel and Schwarz-Linek, 1999; Bocola et al., 2005; Romero et al., 2016).

### ^1^H-NMR Analysis

For ^1^H-NMR analysis, the reactions were carried out at 4 mL for 2-phenylcyclohexanone (10 mM) and 10 mL for benzoin (1.0 mM). Extraction was performed three times with ethyl acetate, dried over anhydrous sulfate magnesium and concentrated by rota-evaporation. The extracts were suspended in 1 mL CDCl_3_ and NMR analysis was performed in a 400 MHz Varian Unity Plus spectrometer.

### Statistical Analysis

All analyses were performed using GraphPad Prism v6.05 for Windows (GraphPad Software, La Jolla, CA, United States). To assess statistical significant differences between more than two groups of data, a two-way ANOVA test was used, with the Tukey post-test used to compare each different group, using a *p* < 0.05. Kinetic parameters were obtained by fitting the obtained data to the Michaelis–Menten equation. Chromatograms and MS spectra were analyzed using GCMSsolution Postrun Analysis 4.11 (Shimadzu). The library for the MS spectra was NIST11.

## Results

### Genome Analysis and Molecular Cloning

By using the sequence motif for Rossmann fold (GxGxxG/A) and two previously described Type I BVMO-specific sequence motifs ([A/G]GxWxxxx[F/Y]P[G/M]xxxD and FxGxxxHxxxWP/D) (Fraaije et al., 2002; Riebel et al., 2012), we could identify three putative BVMOs in the predicted proteome of *S. leeuwenhoekii* C34: Sle_41160, Sle_13190 and Sle_62070 (UniProt codes A0A0F7VV32, A0A0F7VUW7, and A0A0F7W6X7, respectively) (Supplementary Table S2). A sequence alignment analysis revealed that Sle_13190 displays 92% sequence identity with PntE (pentalenolactone D synthase from *S. arenae*) (Seo et al., 2011) while, Sle_62070 only has 50% sequence identity with PntE. Another known BVMO that is closely related in sequence with Sle_62070 is PockeMO (Polycyclic Ketone Monooxygenase, 39% sequence identity) from the fungus *T. thermophila* (Fürst et al., 2016). These two putative BVMOs share 49% of sequence identity. On the other hand, Sle_41160, has 36% sequence identity with HAPMO (4-hydroxyacetophenone monooxygenase from *P. fluorescens*) (Kamerbeek et al., 2001) and shares around 30% sequence identity with the other two predicted BVMOs. A phylogenetic molecular analysis was inferred using ML (Whelan and Goldman, 2001). The resultant tree revealed that Sle_13190 and Sle_62070 belong to a distinct clade of Type I BVMOs (Supplementary Figure S1). Based on the recently elucidated crystal structure of PockeMO, it has been reported that this group of BVMOs have a special structure feature in common that allows them to accommodate relatively large substrates in their active site (Fürst et al., 2016). This suggests that Sle_13190 and Sle_62070 should have the capacity to deal with bulky compounds. On the other hand, Sle_41160 was clustered close to HAPMO, a Type I BVMO described to catalyze the reaction of 4-hydroxyacetophenone to the corresponding acetate ester (Kamerbeek et al., 2001).

The *sle_13190*, *sle_62070*, and *sle_41160* genes have 69, 71, and 73% of G+C% content which is similar to the chromosomal DNA of *S. leeuwenhoekii* (Gomez-Escribano et al., 2015). We amplified the three genes from the isolated genomic DNA after optimizing PCR conditions. Thereupon, we cloned them into a pCRE2 vector that harbors a NADPH-recycling PTDH as a N-terminal fusion partner with a N-terminal histidine tag. The generated expression plasmids were used to transform *E. coli* TOP10, the subsequent expression of soluble protein was tested at various temperatures. The best results for Sle_13190 and Sle_62070 were obtained when expression was performed at 17°C for 48 h using 0.02% of L-arabinose. For Sle_41160, no expression could be obtained at any of the tested conditions and therefore was discarded for further experiments. The proteins were purified through Ni^+2^-affinity chromatography in one step, a clear yellow color was indicative of proper folding and FAD binding. The purified proteins displayed UV-vis spectra that are characteristic for flavin-containing proteins displaying a maxima absorbance at 385 and 440 nm (Supplementary Figure S2). Using SDS as unfolding agent, the extinction coefficient at 450 nm of each flavoprotein was determined: 14.1 and 15.7 mM^-1^ cm^-1^ for Sle_13190 and Sle_62070, respectively.

### Characterization of the Atacama BVMOs

**FIGURE 1:**
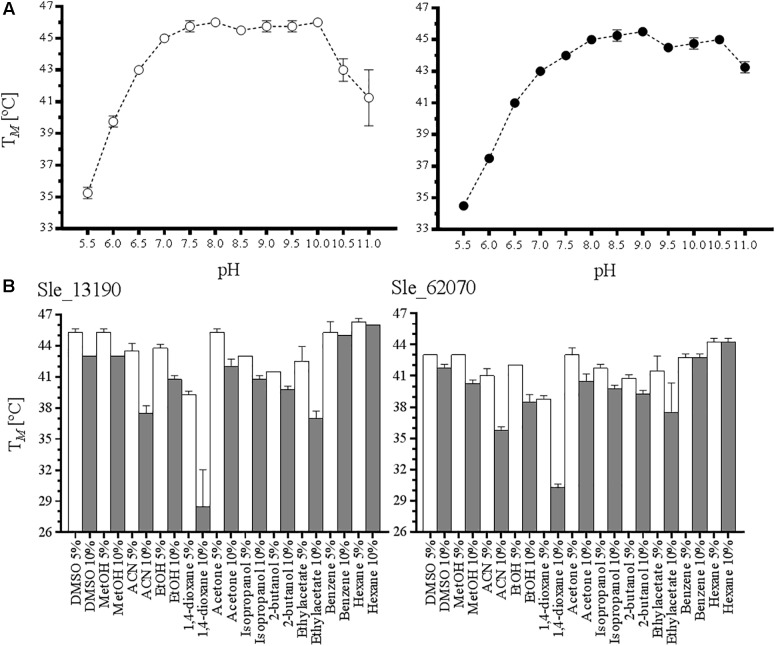
Determination of apparent melting temperature of *S. leeuwenhoekii* Type I BVMOs. **(A)** The thermostability of Sle_13190 (white dots) and Sle_62070 (black dots) were measured at different pH (5.0–11.0). **(B)** The effect in the T*_M_*’ by the presence of cosolvents at 5% v/v (white column) and 10% v/v (black column) was analyzed for Sle_13190 and Sle_62070 in Tris–HCl pH 7.0.

To obtain a better view on the biochemical properties of the two purified flavoenzymes, their thermostability and tolerance toward cosolvents were studied. The ThermoFAD method was used to probe their thermostability. This method determines the apparent melting temperature (T*_M_*’) of a flavoprotein based on the increase in flavin fluorescence when the flavin cofactor is released upon protein unfolding (Forneris et al., 2009). First, we determined the T*_M_*’ of the enzymes at various pH values using different buffers (Tris-HCl, Bis-Tris HCl, or CAPS) in the presence of 100 mM NaCl and 10% w/v glycerol. Interestingly, both flavoproteins display a similar pH-dependent unfolding profile (**Figure 1A**). For Sle_13190 and Sle_62070, the T*_M_*’ was around 45°C between pH 7.5 and 10.0, showing that the two enzymes are relatively thermostable. To discard a possible buffer composition effect, HEPES at pH 7.0–7.5 and citrate buffer at pH 6 were also tested, resulting in highly similar T*_M_*’ values. Subsequently, the effect of several commonly used cosolvents on the thermostability was explored by analyzing samples containing 5 and 10% v/v of DMSO, methanol, acetonitrile (ACN), ethanol, 1,4-dioxane, acetone, isopropanol, 2-butanol, ethyl acetate, benzene or hexane (**Figure 1B**). Again, both flavoenzymes displayed similar patterns of solvent tolerance. For both enzymes, a major deleterious effect was observed with 10% ACN, ethyl acetate and 1,4-dioxane, resulting in a drop of 7–8°C with ACN and ethyl acetate, and a drop of >10°C with 1,4-dioxane. The data suggest that the enzymes can be employed in the presence of various solvents.

The effect of increasing concentrations of NaCl, ectoine, 5-hydroxyectoine and proline was also analyzed. These additives were chosen because the BVMOs may have evolved to operate in the presence of high concentrations of these compounds as *S. leeuwenhoekii* thrives in a highly salty environment. While Sle_13190 was rather insensitive to increasing amounts of NaCl, Sle_62070 was more stable at lower concentrations (Supplementary Figure S3). For the other additives, no significant differences in effects on the T*_M_*’ values were observed. While increasing amounts of ectoine resulted in lower T*_M_*’ values, 5-hydroxyectoine (0–200 mM) and proline (0–4 mM) did not have a significant effect (Supplementary Figure S3).

### Substrate Profiling of the Atacama’s BVMOs

After the thermostability analysis, the substrate scopes for both flavoenzymes were studied through a high-throughput GC-MS analysis approach by verifying product formation for each potential substrate. Each enzyme was incubated with six different mixtures containing 3–6 distinct ketones and thioethers at a final concentration of 400 μM each and 2.5% 1,4-dioxane as cosolvent. In this way, a total of 30 different potential substrates were tested with each BVMO (Supplementary Figure S4). For regeneration of the nicotinamide coenzyme, the fusion partner of the recombinant enzymes, PTDH, was exploited by including phosphite and a catalytic amount of the coenzyme. The conversions were incubated for 24 h at 24°C and after extraction the analysis revealed a broad substrate acceptance for both enzymes. For Sle_13190, 15 compounds were identified as substrate through detection of formed substrate, while Sle_62070 was found to convert 17 of the 30 tested compounds (**Table 1**). The substrate profiles include several cyclic aliphatic ketones, aromatic ketones, and sulfides, linear aliphatic ketones and also a steroid, stanolone. The two BVMOs shared most of the uncovered substrates but also some striking differences were noted. For example, of the tested octanones, Sle_13190 converted 2-octanone, and Sle_62070 converted 3- and 4-octanone (the predicted products are included in the Supplementary Figure S5)

**Table 1:** Substrate scope analysis of Type I BVMOs.

Mix	Substrate	CAS number		Sle_13190	Sle_62070
1	2-hexylcyclopentanone	13074	65-2	+++++	++++
1	3-methyl-2,4-pentanedione	815	57-6	-	-
1	benzylphenyl sulfide	831	91-4	-	+++
1	Cycloundecanone	878	13-7	-	-
1	Indole	120	72-9	-	-
1	Phenylacetone	103	79-7	+++++	+++++
2	2-propylcyclohexanone	94	65-5	+++++	++
2	3-octanone	106	68-3	-	++
2	bicyclo[3.2.0]hept-2-en-6-one	13173	09-6	+++++	+++++
2	Cyclododecanone	830	13-7	-	-
2	Cyclopentanone	120	92-3	-	-
2	methyl-p-tolyl sulfide	1519	39-7	++++	++++
3	2-phenylcyclohexanone	1444	65-1	++++	+++++
3	androsta-1,4-diene-3,17-dione	897	06-3	-	-
3	Cyclopentadecanone	502	72-7	+	+
3	Nicotin	54	11-5	-	-
3	Vanillylaceton	122	48-5	-	-
4	4-hydroxyacetophenone	99	93-4	-	-
4	4-phenylcyclohexanone	4894	75-1	+++++	+++++
4	androst-4-ene-3,17-dione	63	05-8	++++	++
5	4-octanone	589	63-9	-	+
5	Acetophenone	98	86-2	-	-
5	Cyclohexanone	108	94-1	+++	+
5	Pregnenolone	145	13-1	-	-
5	Thioanisole	100	68-5	+++	++++
6	2-octanone	111	13-7	+	-
6	4-methylcyclohexanone	589	92-4	++	+++
6	Benzoin	119	53-9	++	+++++
6	Cyclooctanone	502	49-8	-	-
6	Stanolone	521	18-6	++	+++++


*The 30 compounds tested in the substrate scope analysis are shown with their respective CAS number. Conversion was determined semi-quantitatively for each substrate by analysis of the peak areas in the GC chromatograms normalized by the compound(s) of the mixture that were not accepted by the enzyme. The results are categorized by the observed degree of conversion as 100–81%, +++++; 80–61%; ++++; 60–41%, +++; 40–21%, ++; <20, +; or <1%, -.*

**FIGURE 2:**
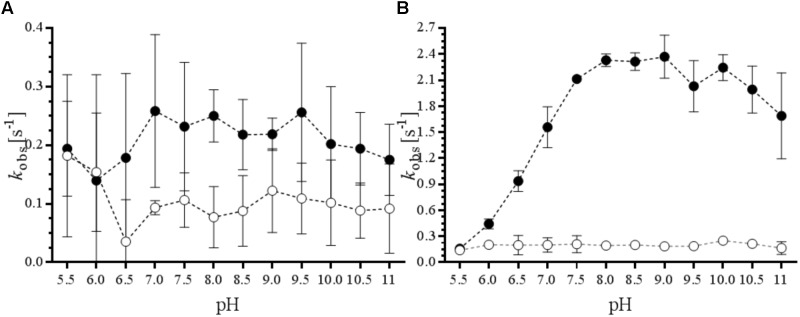
Effects of pH on BVMO and NADPH oxidase activities. The ratio of *k*_obs_ in the presence (black dots) and absence of 5.0 mM phenylacetone (white dots) was analyzed at pH 5.0–11.0 for Sle_13190 **(A)** and Sle_62070 **(B)**.

To determine the optimal conditions and robustness of the potential biocatalysts, phenylacetone was selected as model substrate as it was found to be well-accepted substrate (**Table 1**). We measured the rate of NADPH consumption in the absence or presence of 5.0 mM of this ketone at various pH values (**Figure 2**). The data confirm that Sle_62070 has a good activity with phenylacetone as substrate, and a significantly higher activity with the substrate over the uncoupling activity (consumption of NADPH in absence of substrate). The highest activity (around 2.4 s^-1^) was observed at pH 7.5–10. Sle_13190 has a relatively low activity on phenylacetone (around 0.24 s^-1^ at pH 7–9.5), which is only slightly higher when compared with its uncoupling activity. Based on these activity results we chose a pH value of 8.0 for the subsequent experiments. We also compared the effect of seven water-miscible solvents at 2.5, 5, and 10% v/v (Supplementary Figure S6). This revealed that DMSO is probably a substrate for both BVMOs. For Sle_62070 the rate of NADPH consumption increased significantly at higher DMSO concentrations while for Sle_13190 the observed rate with DMSO were even equal to the rates in the presence phenylacetone. BVMO activity on DMSO has also been noted before and therefore is not a suitable cosolvent (Bordewick et al., 2018). Interestingly, up to 10% v/v of the other six cosolvents seem to be compatible with both BVMOs, even at the highest concentration these cosolvents did not significantly affect the observed activities. While the BVMO activities were in the same range when compared in buffer, only some modest increase in uncoupling activity was seen in the presence of ACN, methanol and isopropanol. This demonstrates that both biocatalysts are rather tolerant toward regularly used cosolvents.

In order to test the effects of known microbial osmoprotectants we studied their effects on Sle_62070 because this BVMO seemed to exhibit better kinetic properties. We chose 1,4-dioxane (2.5% v/v) as cosolvent. The effect of increasing concentrations of ectoin, 5-hydroxyectoine (0–200 mM) and proline (0–4 M) on BVMO activity of Sle_62070 was studied (Supplementary Figure S7). None of the osmoprotectants exerted a dramatic effect on the BVMO or uncoupling activities. A slight boost (20%) on BVMO activity was observed with 200 mM ectoin, while 4 M proline decreased the BVMO activity by 30%. To have a better view on the kinetic properties of Sle_62070, the initial rates of NADPH consumption with a set of ketones and sulfides were determined (Supplementary Figure S8A). These kinetic data revealed a relatively high *k*_obs_ with bicyclo[3.2.0]hept-2-en-6-one, phenylacetone, 2-phenylcyclohexanone and 4-phenylcyclohexanone, and a lower activity with the thioethers methyl p-tolyl sulfide, benzylphenyl sulfide and thioanisole and the cyclic ketones 2-hexylcyclopentanone and cyclopentadecanone. The other tested compounds did not shown a significant difference in activity when compared with the uncoupling rate. The steady-state kinetic parameters for four substrates on which Sle_62070 displayed a relatively high activity were determined (**Table 2**). As it was found for other Type I BVMOs, Sle_62070 displays a relatively high activity with bicyclo[3.2.0]hept-2-en-6-one and phenylacetone, showing a *k_cat_* of 4.0 s^-1^ for both compounds, and *K*_M_ values of 0.2 and 3 mM, respectively (Supplementary Figures S8B,C). In addition, Sle_62070 shows high activity with 2-phenylcyclohexanone and 4-phenylcyclohexanone, displaying *k_cat_* values of >4.0 s^-1^ and *K*_M_ values of >3.0 mM for both phenylcyclohexanones (Supplementary Figures S8D,E). We also attempted to determine kinetic parameters for Sle_13190, but the observed rates were too low for an accurate kinetic analysis. Clearly, using the applied conditions, Sle_62070 is a superior biocatalyst concerning its kinetic properties.

**Table 2:** Kinetic parameters of Sle_62070.

Substrate	*k*_cat_ [s^-1^]	*K*_M_ [mM]	*k*_cat_ *K*_M_^-1^ [s^-1^ mM^-1^]
bicyclo[3.2.0]hept-2-en-6-one	4.0 ± 0.06	0.20 ± 0.01	20
Phenylacetone	4.1 ± 0.2	3.0 ± 0.3	1.3
2-phenylcyclohexanone	>4.0	>3.0	1.3
4-phenylcyclohexanone	>4.0	>3.0	1.3


*NADPH oxidation rates were spectrophotometrically followed in 50 mM Tris–HCl at pH 8.0, 10% glycerol and 100 mM NaCl at 25°C (enzyme 0.45 μM, NADPH 150 μM) with four ketones in increasing concentrations. The data obtained were fit using a Michaelis–Menten equation in GraphPad Prism.*

After establishing the substrate profiles and kinetic properties of the two newly discovered BVMOs, some substrates were selected as candidates to perform conversions at a larger scale. Racemic bicyclo[3.2.0]hept-2-en-6-one was chosen as a hallmark BVMO substrate for enantio- and regioselectivity, phenylacetone as a well described ketone for BVMOs, and thioanisole to include a thioether for testing a sulfoxidation reaction. 2-Phenylcyclohexanone, 4-phenylcyclohexanone and benzoin were selected as relatively unexplored BVMO substrates. All the compounds were tested at 5.0 mM except for the conversion of benzoin; due its poor solubility it was used at a concentration of 1.0 mM. Upon incubating the substrates with 2.7 μM Sle_62070-PTDH for 2 h, complete conversion was observed with most targeted compounds (**Table 3**). For 2-phenylcyclohexanone merely 69% was converted and for thioanisole only 18% conversion was obtained. Extending the incubation to 24 h only resulted in a minor improvement (83 and 22% conversion for 2-phenylcyclohexanone and thioanisole, respectively). Sle_13190 was only tested for the conversion of benzoin, resulting in 40% conversion after 2 h incubation. By GC-MS analysis it was found that both enzymes produce benzaldehyde when converting benzoin. NMR analysis revealed that, except for benzaldehyde, also benzoic acid is formed upon conversion of benzoin (Supplementary Figure S9A). Also the conversion of 2-phenylcyclohexanone was subjected to ^1^H-NMR analysis. The NMR spectral data revealed the production of the proximal lactone when 2-phenylcyclohexanone was converted by Sle_62070 (Supplementary Figure S9B).

**Table 3:** Biocatalysis performance of Sle_62070.

Bicyclo[3.2.0]hept-2-en-6-one	*ee*_N_	*ee*_A_	Phenylacetone	2-phenylcyclohexanone	4-phenylcyclohexanone	Benzoin	Thioanisole
>99%	>99%	>99%	>99%	69%	>99%	>99%	18%


*Biotransformation assays were carried out for 2 h at 24°C for six different substrates, consumption percentages are shown. Enantioselectivity was determined for bicyclo[3.2.0]hept-2-en-6-one. N:A, ratio normal and abnormal product, ee, enantiomeric excess of the product calculated as $ee=\frac{\left(A_{major}-A_{\mathrm{minor}}\right)}{\left(A_{major}-A_{\mathrm{minor}}\right)}\times 100$*

To probe the enantioselectivity of Sle_62070, chiral GC analyses were performed. First, the conversion of 4-phenylcyclohexanone was studied. As reference the conversion of 4-phenylcyclohexanone with TmCHMO-PTDH was performed. The reaction using CHMOs is described to produce preferably the *S* lactone (Vogel and Schwarz-Linek, 1999; Rial et al., 2008). The results revealed that Sle_62070 is highly enantioselective toward this ketone as only the *S* lactone was formed (Supplementary Figure S10). Using 2-phenylcyclohexanone as racemic substrate, Sle_62070 was found to be highly enantioselective for convert the *R* enantiomer substrate. The reaction was compared with TmCHMO-PTDH which is described to display a higher preference for the same enantiomer (Alphand et al., 1996; Bocola et al., 2005; Supplementary Figure S10). The enantioselective properties of Sle_62070 with racemic bicyclo[3.2.0]hept-2-en-6-one as substrate was also analyzed. As reference reaction, the biotransformation of the racemic prochiral cyclic ketone was also performed with PAMO-PTDH resulting in the formation of all four possible lactone products (Romero et al., 2016). After 2 h conversion, two of the four possible lactone products, in equal amounts, were observed when using Sle_62070: the (-)-1*S*,5*R* normal lactone and the (-)-1*R*,5*S* abnormal lactone. The enantiomeric excess for both products were determined as >99% (Supplementary Figure S11). Finally, the reaction was analyzed in time (Supplementary Figures S12A,B) which revealed that Sle_62070 has no preference for one of the two substrate enantiomers.

## Discussion

**FIGURE 3:**
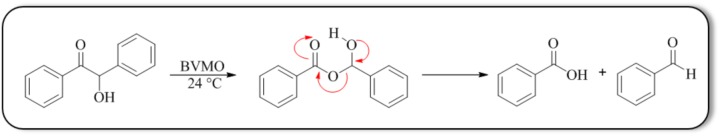
Proposed reaction of benzoin oxidation by Sle_62070. Mechanism of the hydrolysis of oxidation product to benzaldehyde and benzoic acid.

By genome sequence analysis, we have identified two new actinobacterial BVMOs. As far as we know, these are the first BVMOs described from an Atacama desert’s microorganism. Both BVMOs were shown to be rather robust by tolerating cosolvents up to 10% v/v and by displaying relatively high melting temperatures. Compared with CHMO from *Acinetobacter* sp. or STMO from *Rhodococcus rhodochrous* (both having a T*_M_*’ of 39°C), these two new BVMOs showed a higher T*_M_*’ (5–6°C higher). The two BVMOs are similar in thermostability when compared with the recently reported PockeMO (T*_M_*’ of 47°C) which was identified from a thermophilic fungus (Fürst et al., 2016). Both uncovered enzymes showed activity on a wide variety of ketones and sulfides, a typical feature of Type I BVMOs. As already can be deduced from the clustering of the sequences based on sequence homology with other BVMOs that are known to act on bulky compounds, Sle_13190 and Sle_62070 also accept rather complex compounds as substrate, including biphenyls and a steroid. Remarkably, even though the substrate scope is similar between these two enzymes, Sle_62070 showed to be more promising by acting on more compounds and by exhibiting higher activities. Sle_62070 also revealed to be highly enantio- and regioselective in converting bicyclo[3.2.0]hept-2-en-6-one into two enantiopure lactones. Interestingly, it is also efficient in converting benzoin which, as far we know, was only reported as substrate for CPMO without any product identification (Riebel et al., 2012). We have identified for the first time the products formed by BVMO-catalyzed benzoin oxidation: benzaldehyde and benzoic acid. For the production of these compounds, we suggest the formation of a labile ester product which decomposes to from the two aromatic products (**Figure 3**). Overall, this work has delivered two new BVMOs to complement the available collection of known BVMOs. The extreme environment of the Atacama desert may develop as an interesting source for new robust enzymes. Using metagenomic approaches it should become feasible to tap novel biocatalysts from this rich source of actinobacterial “biosynthetic dark matter,” which is unique due to the special soil subsurface geochemistry, ecological diversity, and environmental conditions of this hyper-arid desert (Bull et al., 2016; Idris et al., 2017).

## Author Contributions

AG-S, LP, and MF conceived and designed the study, analyzed the data, and prepared the manuscript. AG-S performed the experiments. MT analyzed the ^1^H-NMR results.

## Conflict of Interest Statement

The authors declare that the research was conducted in the absence of any commercial or financial relationships that could be construed as a potential conflict of interest.

## Footnotes

1 www.genome.jp/tools/motif/MOTIF2.html

2 www.uniprot.org

3 blast.ncbi.nlm.nih.gov/Blast.cgi
