# Supplementary material for: Mining the Genome of Streptomyces leeuwenhoekii: Two New Type I Baeyer–Villiger Monooxygenases From Atacama Desert
Source: Front Microbiol. 2018 Jul 18;9:1609. doi: 10.3389/fmicb.2018.01609 (PMC6058054; doi:10.3389/fmicb.2018.01609)
Supplement: Supplementary file 1 [file Data_Sheet_1.docx]

Supplementary Material

Mining the Genome of *Streptomyces leeuwenhoekii*: Two new Type I Baeyer-Villiger Monooxygenases from Atacama Desert

**Alejandro Gran-Scheuch^1,2^, Milos Trajkovic^1^, Loreto Parra^2,3*^, Marco W. Fraaije^1*^**

^1^Molecular Enzymology Group, University of Groningen, Groningen, The Netherlands.

^2^Department of Chemical and Bioprocesses Engineering, School of Engineering, Pontificia Universidad Católica de Chile, Santiago, Chile

^3^Institute for Biological and Medical Engineering, Schools of Engineering, Medicine and Biological Sciences, Pontificia Universidad Católica de Chile, Santiago, Chile.

**Correspondence:**
^*^Corresponding Author

[m.w.fraaije@rug.nl](mailto:m.w.fraaije@rug.nl)

[lparrat@uc.cl](mailto:lparrat@uc.cl)

**Content**

1. Supplementary table…………………………………………………………………………...2
2. Supplementary figures…………………………………………………………………………4

# Supplementary tables

# Table S1. Gas chromatography parameters

| Analysis | Column | GC program | | | Split ratio | Injection [µL] |
| --- | --- | --- | --- | --- | --- | --- |
|  |  | Rate [°C min^-1^] | Temperature [°C] | Hold time [min] |  |  |
| Racemic bicycle[3.2.0]hept-2-en-6-one | CP Chiralsil Dex CB Agilent, 25 m x 0.25 mm x 0.25 μm | - | 40 | - | 50.0 | 3 |
|  |  | 10 | 130 | 15 |  |  |
|  |  | 10 | 40 | - |  |  |
| 4-Phenylcyclohexanone |  | -  10  1  10 | 80 | - | 50.0 | 3 |
|  |  |  | 110 | - |  |  |
|  |  |  | 200 | 20.00 |  |  |
|  |  |  | 80 | - |  |  |
| 2-Phenylcyclohexanone |  | - | 40 | - | 20.0 | 1 |
|  |  | 10 | 180 | 0 |  |  |
|  |  | 1 | 200 | 15 |  |  |
|  |  | 10 | 40 | 0 |  |  |
| Phenylacetone | HP-1 Agilent, 30 m x 0.25 mm x 0.25 μm | - | 30.0 | 5.00 | 5.0 | 2 |
|  |  | 5.00 | 70.0 | 5.00 |  |  |
|  |  | 5.00 | 130.0 | 10.00 |  |  |
| 2-Phenylcyclohexanone  4-Phenylcyclohexanone |  | - | 30.0 | 5.00 | 10.0 | 2 |
|  |  | 10.00 | 160.0 | 5.00 |  |  |
|  |  | 5.00 | 250.0 | 5.00 |  |  |
| Benzoin |  | - | 55.0 | 5.00 | 5.0 | 2 |
|  |  | 5.00 | 78.0 | 3.00 |  |  |
|  |  | 10.00 | 170.0 | 1.00 |  |  |
|  |  | 5.00 | 180.0 | 3.00 |  |  |
|  |  | 5.00 | 215.0 | 5.00 |  |  |
|  |  | 10.00 | 250.0 | 3.00 |  |  |
| Substrate scope analysis |  | - | 30 | 5 | 5.0 | 2 |
|  |  | 5 | 70 | 5 |  |  |
|  |  | 5 | 130 | 5 |  |  |
|  |  | 10 | 325 | 5 |  |  |

| Predicted protein | Uniprot Accesion number | Rossman motif | Type I BVMO fingerprints | | Rossman motif | 90% identity Cluster name |
| --- | --- | --- | --- | --- | --- | --- |
|  |  | G-x-G-x-x-[G/A] | [A/G]-G-x-W-x-x-x-x-[F/Y]-P-[G/M]-x-x-x-D | F-x-G-x-x-x-H-x-x-x-W-[P/D] | G-x-G-x-x-[G/A] |  |
| Sle_13190 | A0A0F7VUW7 | **G**A**G**IG**G** | **GG**T**W**YWNR**FPG**VRC**D** | **F**A**G**HSF**H**TSR**WD** | **G**T**G**ST**T** | Pentalenolactone D synthase |
| Sle_62070 | A0A0F7W6X7 | **G**G**G**FG**G** | **GG**T**W**YWNR**YPG**IHC**D** | **F**E**G**HTF**H**TSR**WD** | **G**T**G**AT**G** | Phenylacetone monooxygenase |
| Sle_41160 | A0A0F7VV32 | **G**S**G**FG**G** | **GG**T**W**RDNS**YPG**CAC**D** | **F**P**G**KVF**H**SAR**WD** | **G**T**G**AS**A** | Uncharacterized monooxygenas |

**Table S2. Identification of putative Type I BVMO in *S. leeuwenhoekii* proteome***.* The genome analyses are shown in the table with the predicted proteins, their respective Uniprot accession number, the predicted amino acid sequence of the conserved motif (two Rossman and the Type I BVMO fingerprints) and the names of the cluster judged by percentage of identity.

# Supplementary Figures

**

**

**Figure S1. Molecular phylogenetic analysis.** The evolutionary history was inferred by using the Maximum Likelihood method; the tree with the highest log likelihood (-14431,27) is shown. The tree is drawn to scale, with branch lengths measured in the number of substitutions per site. The analysis involved 45 amino acid sequences including CDMO (cyclododecanone monooxygenase), CPDMO (cyclopentanone monooxygenase), PockeMO (polycyclic ketone monooxygenase), CPMO (cyclopentanone monooxygenase), MEKMO (methylethylketone monooxygenase), ACMO (acetone monooxygenase), PAMO (phenylacetone monooxygenase), SAPMO (4-sulfoacetophenone monooxygenase) STMO (steroid monooxygenase), OTEMO (2-oxo-Δ^3^-4,5,5- trimethylcyclopentenylacetyl-CoA monooxygenase), CHMO (cyclohexanone monooxygenase), HAPMO (4-hydroxyacetophenone monooxygenase), EthA (Ethionamide monooxygenase), pentalenolactone biosynthetic genes PenE, PntE and PtlE, 24 BVMOs from *Rhodococcus jostii* RHA1 and the three putative proteins from *S. leeuwenhoekii* C34*.*

**
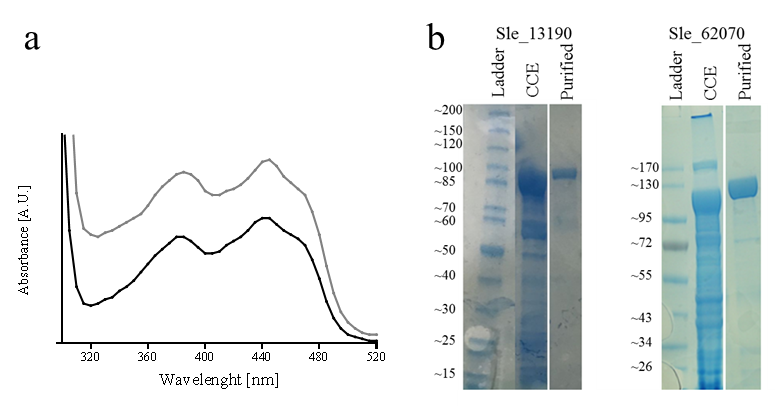
**

**Figure S2. Purification of Sle_13190 and Sle_62070.** (a) UV-Vis absorbance spectra of purified Type I BVMOs; Sle_13190 in black and Sle_62070 in gray. (b) SDS-PAGE of both purified flavoproteins and their respective clarified crude extracts.

**Figure S3**. **Determination of apparent melting temperatures (T*_M_***’**) of *S. leeuwenhoekii* Type I BVMOs with additives.** The T*_M_*’ of Sle_62070 (gray column) and Sle_13190 (white column) were determined using increasing concentrations of NaCl, ectoine, 5-hydroxyectoine and proline as additives, n=3.


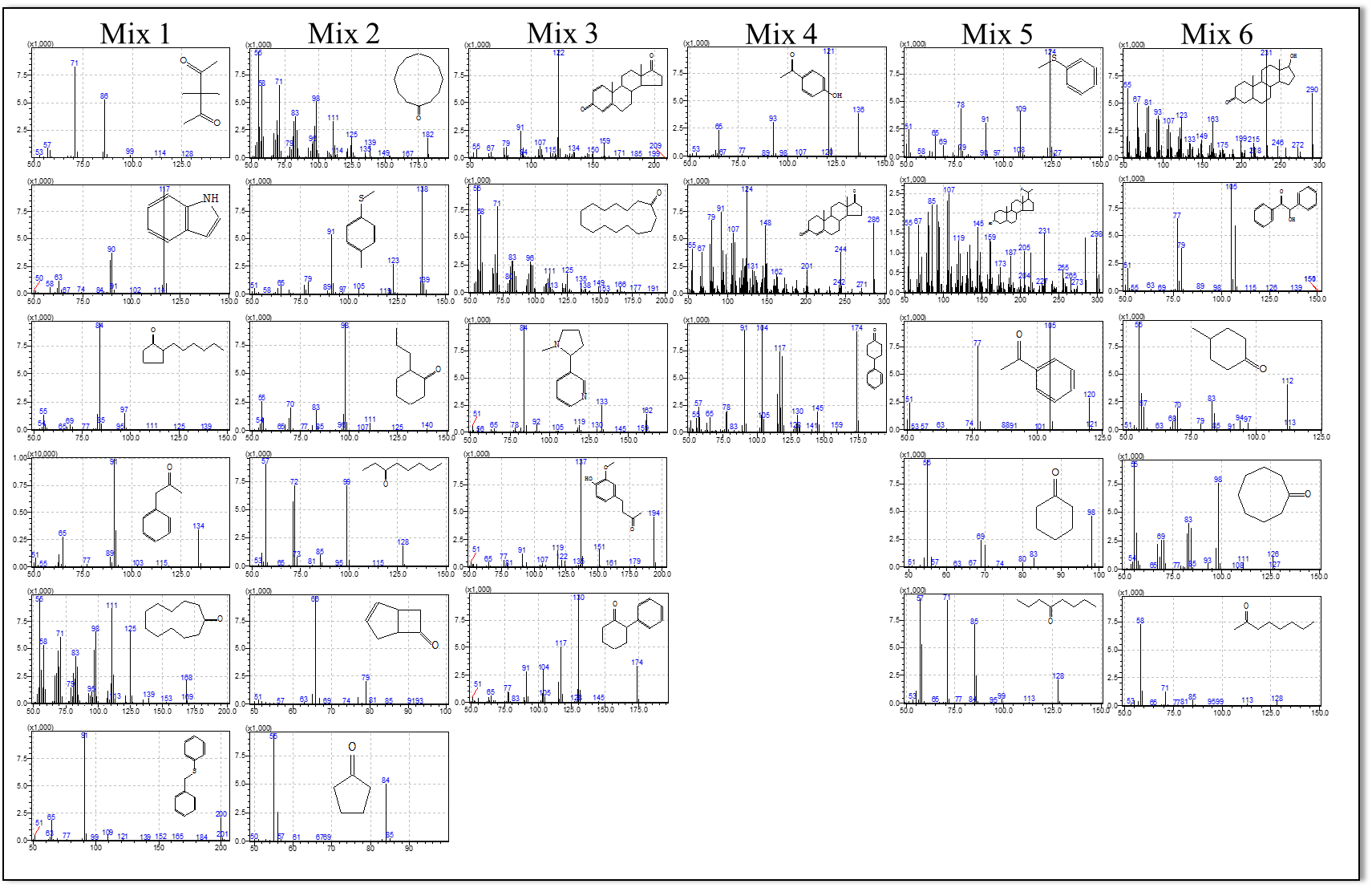


**Figure S4**. **List of compounds used for substrate scope analysis.** 30 Compounds were used in the analysis of substrate acceptance using 6 different compound mixtures. The composition of the mixtures are shown in columns with the structure and the respective ms spectrum obtained from GCMSsolution Postrun Analysis 4.11 (Shimadzu). Mix 1,­ 3-methyl-2,4-pentanedione, indole, 2-hexylcyclopentanone, phenylacetone, cycloundecanone and benzylphenyl sulfide; mix 2, cyclododecanone, methyl-p-tolyl sulfide, 2-propylcyclohexanone, 3-octanone, biclye[3.2.0]hept-2-en-6-one and cyclopentanone; mix 3, androst-1,4-diene-3,17-dione, cyclopentadecanone, nicotine, vanillylacetone and 2-phenylcyclohexanone; mix 4, 4-hydroxyacetophenone, androst-4-ene-3,17-dione, 4-phenylcyclohexanone; mix 5, thioanisole, pregnalone, acetophenone, cyclohexanone and 4-octanone; mix 6, stanolone, benzoin, 4-methylcyclohexanone, cyclooctanone and 2-octanone.

| **Substrate** | **Product** | **MS spectra** |
| --- | --- | --- |
| **** | **** | **** |
| **** | **** | **** |
| **** | **** | **** |
| **** | **** | **** |
| **** | **** | **** |
| **** | **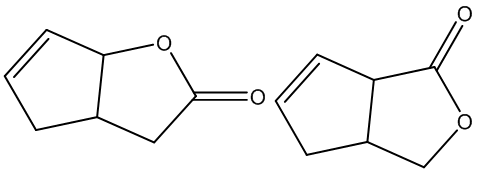** | **** |
| **** | **** | **** |
| **** | **^*^** | **** |
| **** | **** | **** |
| **** | **^*^** | **** |
| **** | **** | **** |
| **** | **** | **** |
| **** | **** | **** |
| **** | **** | **** |
| **** | **** | **** |
| **** | **** | **** |
| **** | **^*^** | **** |
| **** | **** | **** |

**^*^**The products of 2-phenylcyclohexanone and benzoin were analyzed by ^1^H-NMR. While the product for 4-phenylcyclohexanone was not in the MS spectrum library: the molecular structure depicts the expected BV product.

**Figure S5. Lists of substrate and products, together with MS fragmentation patterns of the products.**


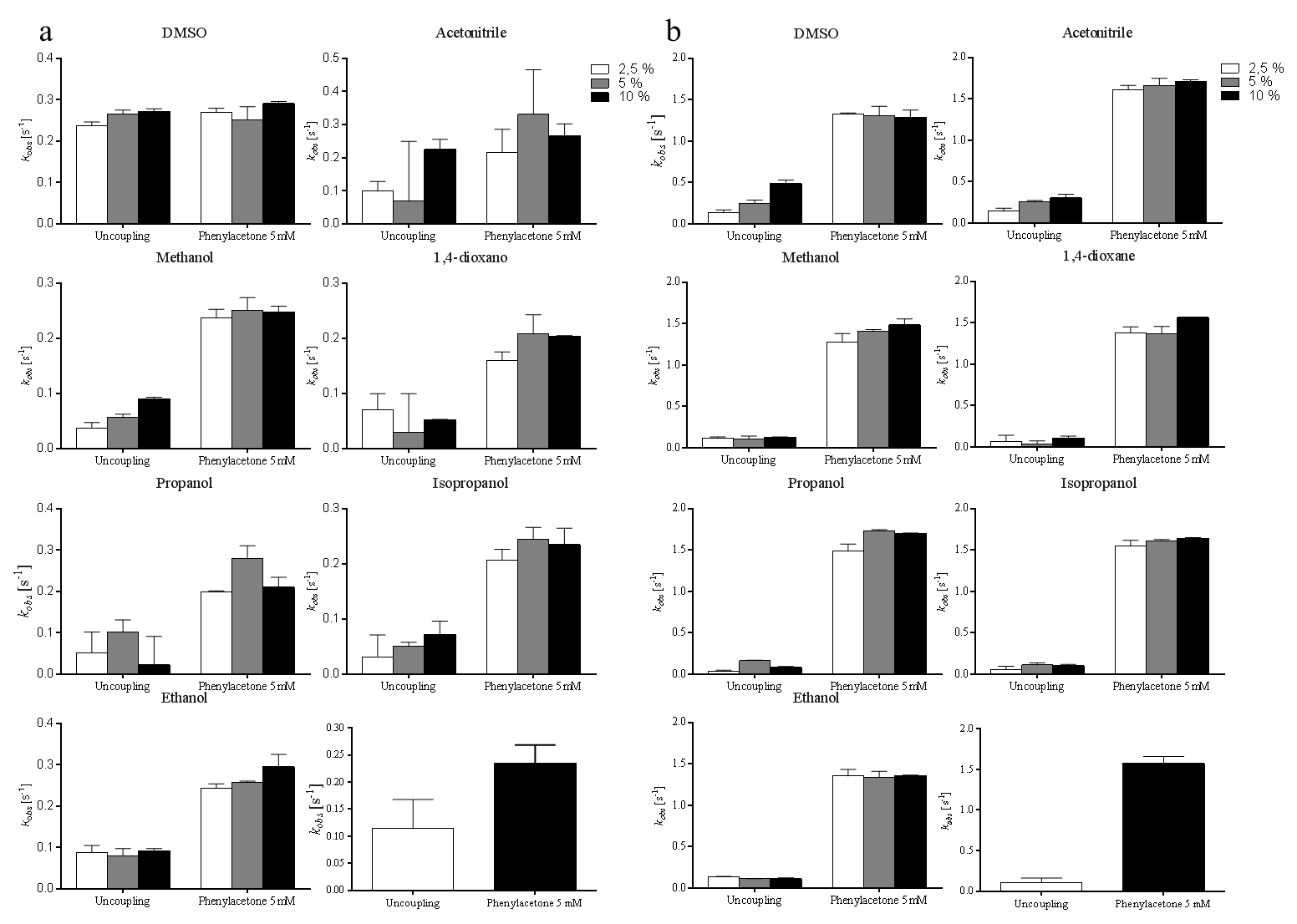


**Figure S6**. **Effects of water-miscible cosolvents in BVMO and NADPH oxidase activity.** Activity (*k*_obs_) was measured using 5.0 mM phenylacetone or in the absence of substrate [(a) Sle_13190 and (b) Sle_62070]. The effect of cosolvent was tested using 2.5 (white column), 5 (gray column) and 10 % v/v (black column) of the cosolvent (DMSO, acetonitrile, methanol, 1,4-dioxane, propanol, isopropanol or ethanol), n=3.

**
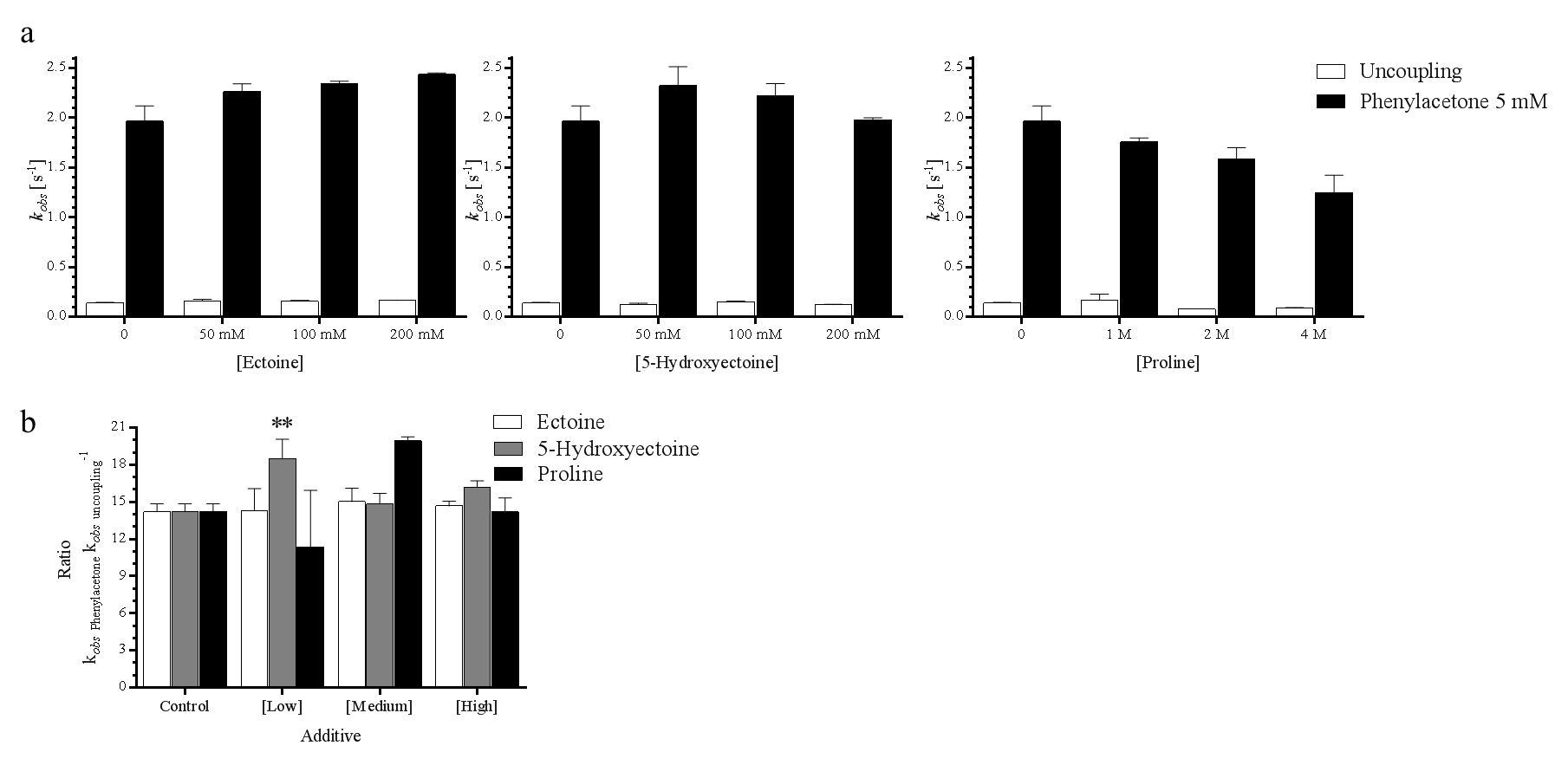
**

**Figure S7. Effect of additives in BVMO and NADPH oxidase activity of Sle_62070.** (a) The *k_obs_* of Sle_62070 in presence of 5.0 mM phenylacetone (black column) and in absence of substrate (white column) was measured at increasing concentrations of ectoine, 5-hydroxyectoine and proline. (b) The ratio *k*_obs_ with phenylacetone and in absence of substrate was calculated for the additive conditions using ectoine (white column), 5-hydroxyectoine (gray column) and proline (black column), n=3, **=statistical significance with *p* < 0.05.

**
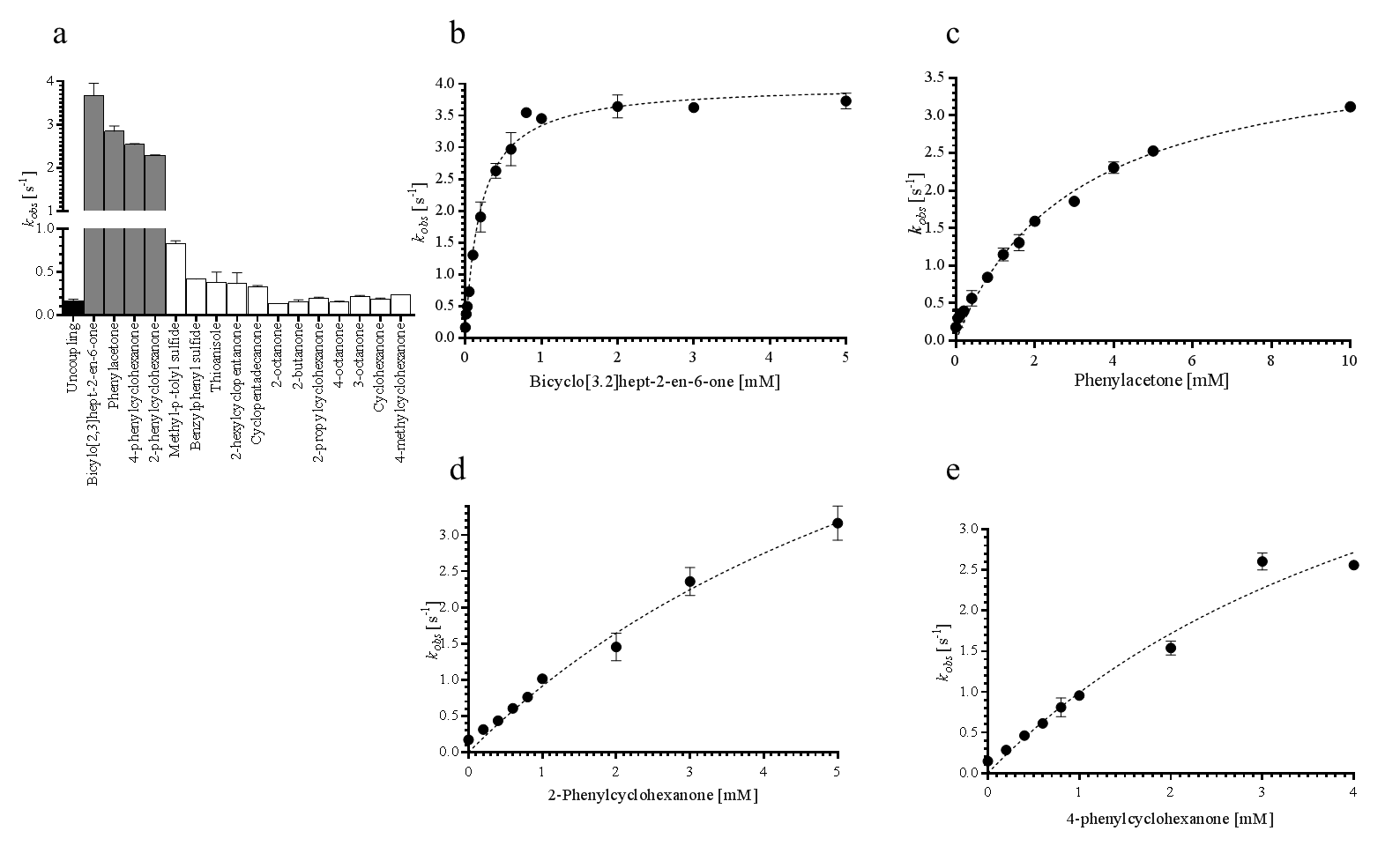
**

**Figure S8. NADPH oxidation activity analysis of Sle_62070.** (a) NADPH oxidation was spectrophotometrically followed at 340 nm using Sle_62070 as catalyst with different ketones and sulfides. As a control the uncoupling rate was measured (black column). The substrates that showed highest activity (gray columns) were selected for a more detailed kinetic analysis: (b) bicyclo­[3.2.0]­hept-2-en-6-one, (c) phenylacetone, (d) 2-phenylcyclohexanone and (e) 4-phenyl­cyclo­hexanone, n=3.


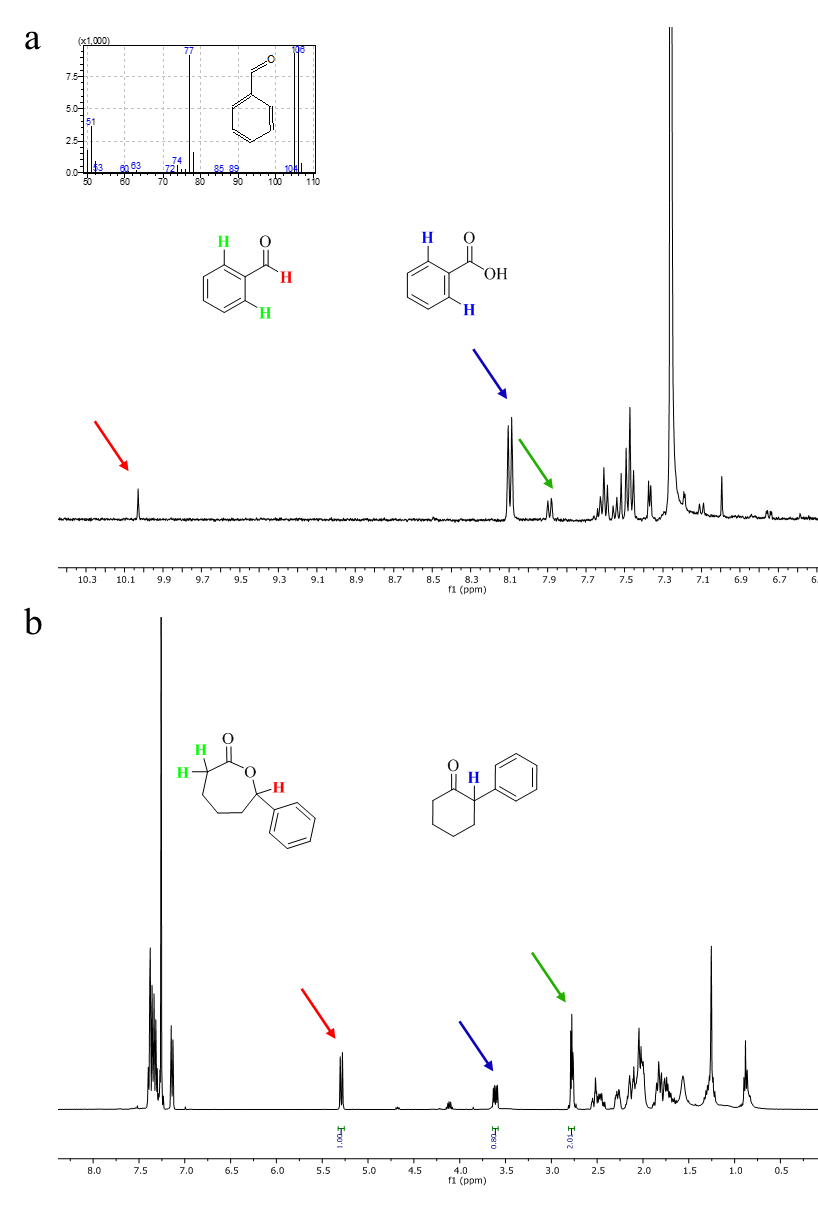


**Figure S9. Identification of reactions products using Sle_62070 as biocatalyst.** (a) ^1^H-NMR analysis in CDCl_3_ at 400 MHz of reaction with benzoin as substrate and MS spectrum of benzaldehyde obtained in GC-MS. (b) ^1^H-NMR analysis of 2-phenylcyclohexanone reaction in CDCl_3_ at 400 MHz.


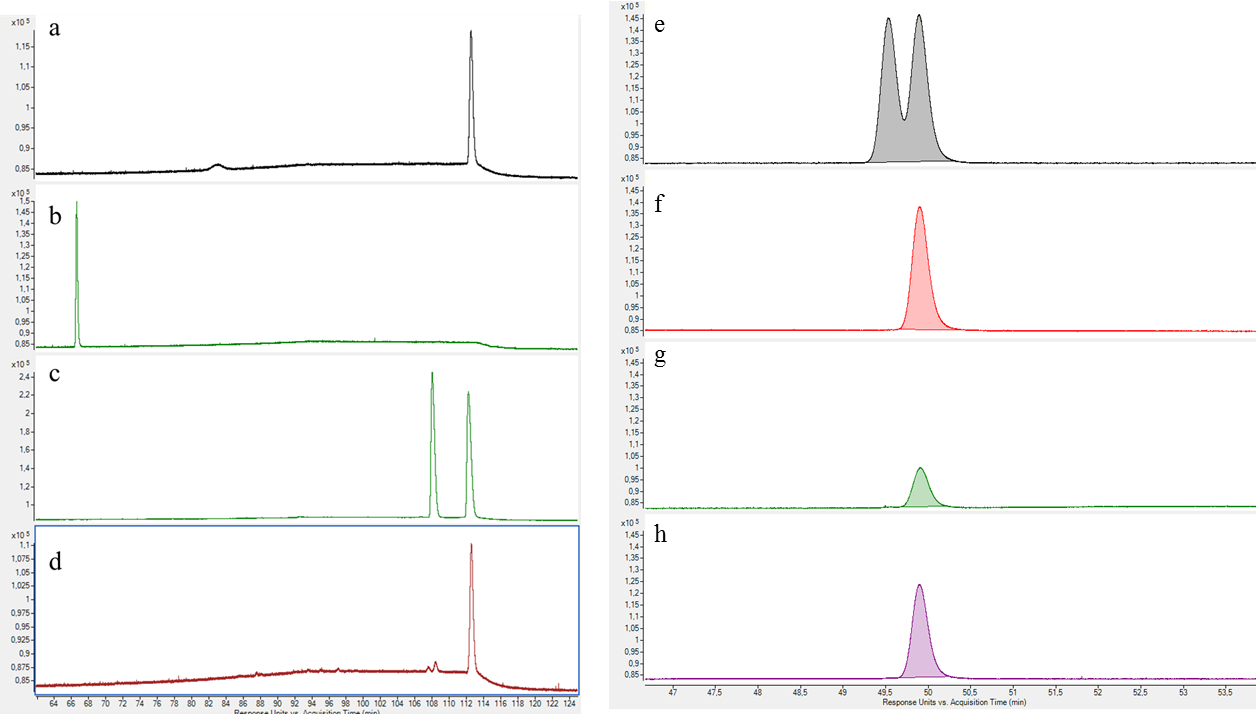


**Figure S10. Biotransformation of Sle_62070 using 2- and 4-phenylcyclohexanone.** Reactions contained purified enzyme (2.7 μM), 2-phenylcyclohexanone or 4-phenylcyclohexanone (5.0 mM), NADPH (150 μM), Na_2_PO_3_•5H_2_O (10 mM), FAD (30 μM), NaCl (100 mM), glycerol (10 % w/v) in 50 mM Tris-HCl at pH 8.0 and 1,4-dioxane as cosolvent (2.5 % v/v). For (a) 4-phenylcyclohexanone reactions were analyzed by chiral GC after 2 h at 24 °C. (b) Substrate and (c) synthetized racemic products were also analyzed. Chromatograms were compared with (d) TmCHMO-PTDH described to produce preferably the *S* lactone. For 2-phenylcyclohexanone reactions were analyzed after (e) 0, (f) 2 and (g) 24 h at 24 °C. Chromatograms were compared with (h) TmCHMO-PTDH described to display *R* selectivity.


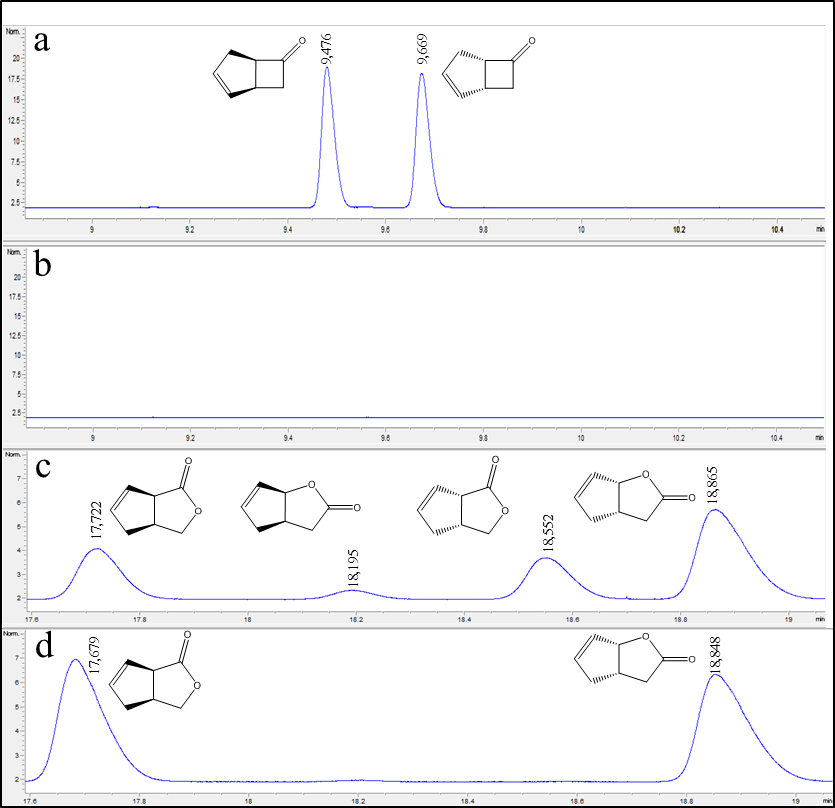


**Figure S11. Chromatograms of the biotransformation using Sle_62070**. Reactions contained purified enzyme (2.7 μM), *rac*-bicyclo[3.2.0]hept-2-en-6-one (5.0 mM), NADPH (150 μM), Na_2_PO_3_•5H_2_O (10 mM), FAD (30 μM), NaCl (100 mM), glycerol (10 % w/v) in 50 mM Tris-HCl at pH 8.0 and 1,4-dioxane as cosolvent (2.5 % v/v). Reactions containing rac-bicyclo[3.2.0]hept-2-en-6-one were analyzed by chiral GC after (a) 0 and (b & d) 2h at 24 °C. Sle_62070 fully converted *rac*-bicyclo[3.2.0]hept-2-en-6-one and produced almost exclusively one regioisomer from each enantiomer, namely (1S,5R)-2-oxabicyclo[3.3.0]oct-6-en-3-one (normal product) and (1R,5S)-3-oxabicyclo[3.3.0]oct-6-en-2-one (abnormal product). Retention times were compared with (c) where PAMO-PTDH was used for converting *rac*-bicyclo[3.2.0]hept-2-en-6-one and which is known to yield all four possible lactones.

**
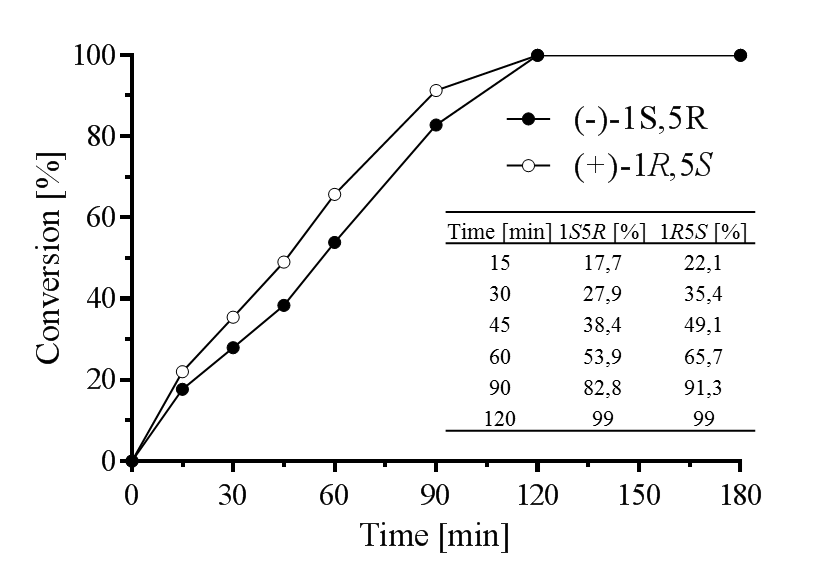
**

a

b

**
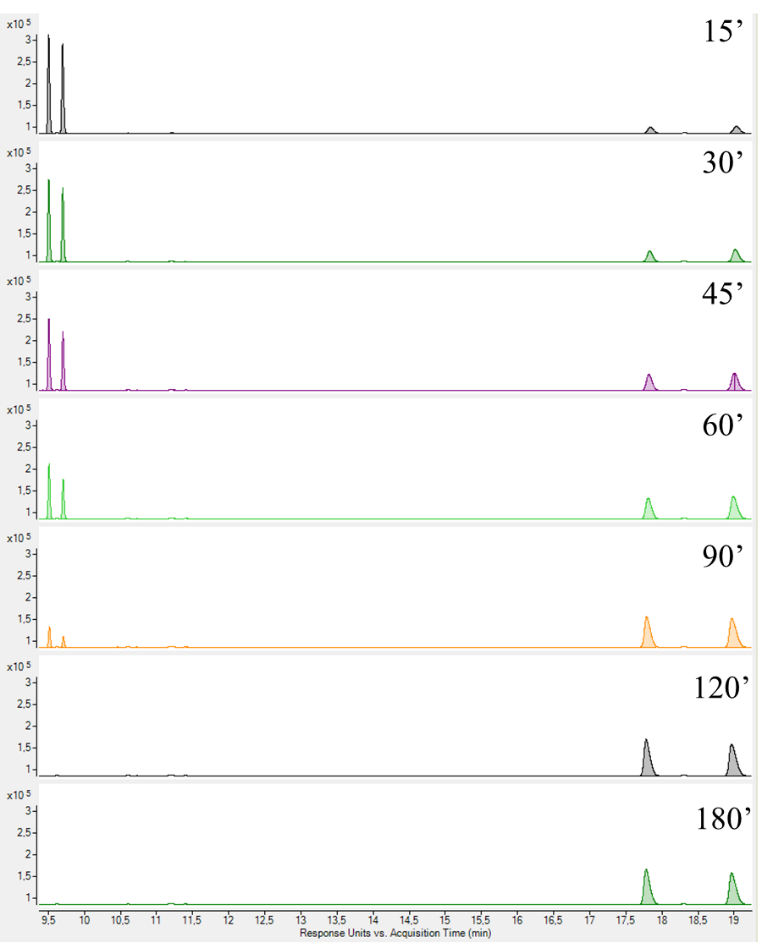
**

**Figure 12. Biotransformation of *rac*-bicyclo[3.2.0]hept-2-en-6-one in time.** Biotransformations using Sle_62070 and *rac*-bicyclo[3.2.0]hept-2-en-6-one were followed in time. (a) Conversion of (-)-1*S*,5*R* (black) or (+)-1*R*,5*S* (white) substrate are shown in time. (b) Chromatograms of time point reaction are shown (15, 30, 45, 60, 90, 120 and 180 min). Retention time of substrates (-)-1*S*,5*R,* (+)-1*R*,5*S* and the products (abnormal and normal) were 9.47, 9.67, 17.68 and 18.85, respectively.
